# The use of virtual reality to reduce stress among night-shift anesthesiologists: study protocol for a crossover trial

**DOI:** 10.1186/s13063-021-05222-8

**Published:** 2021-04-07

**Authors:** Chaojin Chen, Liubing Chen, Ning Shen, Chenfang Luo, Ren Wang, Hongyi Fang, Qi Zhang, Ziqing Hei

**Affiliations:** 1 Department of Anesthesiology, The Third Affiliated Hospital of Sun Yat-sen University, No. 600 Tianhe Road, Guangzhou, 510630 Guangdong Province China; 2 Cell-gene Therapy Translational Medicine Research Center, The Third Affiliated Hospital of Sun Yat-sen University, No. 600 Tianhe Road, Guangzhou, 510630 Guangdong Province China

**Keywords:** Virtual reality, Anesthesiologists, Stress, Work overload, Night shift

## Abstract

**Background:**

Because of the lack of anesthesia workforce, anesthesiologists are forced to work overtime and more night shifts, which can disturb their biological rhythm and cause severe stress and depression, potentially leading to negative and even devastating outcomes for both themselves and patients. Virtual reality (VR), a new method to reduce stress and pain for patients, has been widely used in biomedical fields. The purpose of this study is to evaluate the potential effectiveness of VR technology in reducing stress among night-shift anesthesiologists.

**Methods:**

In this randomized controlled, crossover, single-center clinical trial, a total of 30 anesthesiologists will be enrolled and randomized in a 1:1 allocation to either the VR immersion group (intervention group) or the routine night-shift group (control group) with a washout of 1 week. Anesthesiologists in the intervention group will undergo VR immersion twice, while anesthesiologists in the control group will not watch VR videos during the night shift. The primary outcome will be the difference in the NASA Task Load Index (NASA-TLX) score between the two groups. Secondary outcomes will include the Chinese Perceived Stress Scale (CPSS), perceived stress scores (visual analogue scale (VAS)), and Multidimensional Fatigue Inventory (MFI-20) scores; levels of satisfaction among the participants; incidence of arrhythmia; and incidence of chest tightness, headache, and palpitations.

**Discussion:**

It is unknown whether the use of VR technology during the night shift can reduce stress among anesthesiologists. With the widespread use of VR technology, a positive result in this trial could spur hospitals to apply VR technology to reduce stress among night-shift doctors in every department and provide a relatively relaxed working environment.

**Trial registration:**

Chinese Clinical Trial Registry ChiCTR2000031025. Registered on 21 March 2020

## Background

More than 300 million people need to undergo surgery each year worldwide [1]; more than 70% of those people have no access to safe, affordable anesthesia or surgical care [2], and the severe lack of anesthesiologists is one of the causes of this phenomenon [3]. The lack of first-line doctors also leads to more work overload and night shifts, which disturbs the biological rhythm and causes severe stress and depression [4, 5]. Due to long working hours, work overload, chronic sleep deprivation, the need for sustained vigilance, continuous noise pollution and even halitosis among patients [6], and the risk of COVID-19 [7], anesthesiologists face high levels of job-related stress [8, 9], which causes anxiety, depression, sleep disturbances, memory and attention problems, nightmares, the need for medication, and so on [10, 11]. Lindfors et al. [12] showed that approximately 68% of Finnish anesthesiologists felt stress at work, and the proportion was 79% in Ireland [13]. It has been reported that anesthesiologists suffer higher levels of anxiety and stress than those in other occupations [14]. When anesthesiologists are under high levels of stress for a long time, there may be negative or even devastating outcomes for the patients. Therefore, an intervention that could reduce stress among night-shift anesthesiologists could be valuable in enhancing medical safety and protecting the health of the anesthesiologists.

Recently, virtual reality (VR) has been widely used in biomedical fields [15]. It can alter our sense of personal presence to that of being in a virtual world; therefore, the features of sensory and affective experience can be changed. This new technology has been applied in hospitals to create an immersive environment to minimize stress for both patients and healthcare providers [16–18]. For instance, VR has been widely used before surgery to reduce preoperative anxiety (measured using visual analogue scales (VAS)) in patients [19]. Moreover, the broad reach of VR has enabled its use for treating pain, psychological stress, social and generalized anxiety disorders, depression, and posttraumatic stress disorder (PTSD) as well as helping with poststroke rehabilitation [20]. In addition, VR technology had been used by surgeons in clinical work to decrease the physical and mental workload [21] and reduce the stress response and cognitive load in inpatient oncology nurses [16]. However, few studies have focused on the application of VR technology to reduce stress among night-shift anesthesiologists. Therefore, we conducted this study to investigate the potential effectiveness of VR technology in reducing stress among night-shift anesthesiologists.

## Methods/design

### Study design

**Fig. 1:**
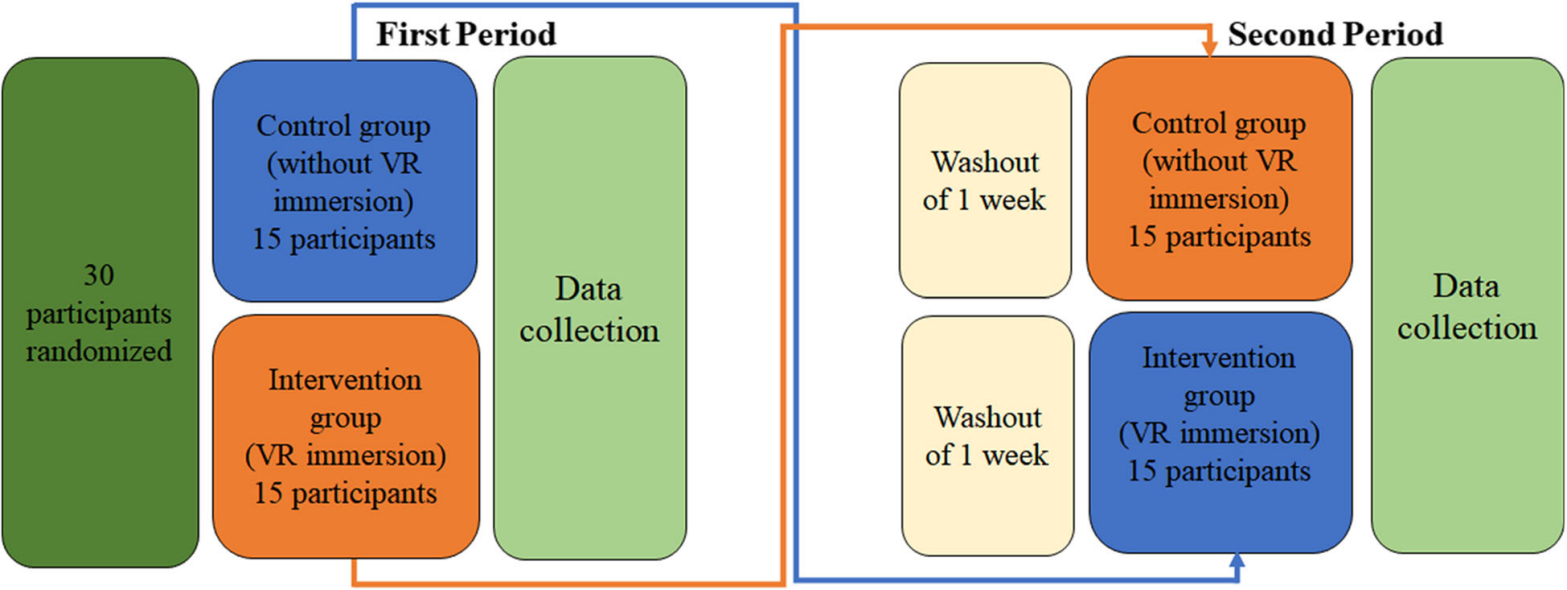
The crossover design of the study

The virtual reality immersion in the night-shift anesthesiologist trial is a randomized controlled, single-center clinical trial using a crossover design with a washout of 1 week. Anesthesiologists will be randomized in a 1:1 allocation to a sequence of either immersion relaxation using VR in the first period, followed by no immersion VR in the second treatment period, or no immersion VR in the first period, followed by immersion relaxation using VR in the second period. Anesthesiologists undergoing VR intervention will undergo immersion relaxation via VR twice during the night shift: the first session will occur at 23:30, and the second session will occur at 07:30 the next day. The anesthesiologists will be able to choose VR content and enjoy themselves for 20 min. Anesthesiologists in the control group will not watch VR videos during the night shift. The design of the study is presented in Fig. 1.

The primary outcome of the study will be the NASA Task Load Index (NASA-TLX) at 07:50 of the next day, which consists of six dimensions rated on a 100-point scale in each dimension (physical demand, mental demand, temporal demand, effort, performance, and frustration) and is widely used to measure the mental workload [22, 23]. Secondary outcomes will be the Chinese Perceived Stress Scale (CPSS), perceived stress scores (visual analogue scale (VAS)), and Multidimensional Fatigue Inventory (MFI-20) scores; the level of satisfaction among participants; heart rate (HR); blood pressure (BP); and incidence of arrhythmia, chest tightness, headache, and palpitations at all the recorded timepoints. The CPSS was used to assess the severity of participants’ psychological stress as earlier reported [24]. Perceived stress score was assessed via the VAS score, which was defined as the length in millimeters from 0 (no stress) to 10 (unbearable stress) [25]. The MFI-20 score was used to quantify the impact of fatigue [26], including five independent subscales of fatigue: general fatigue, mental fatigue, physical fatigue, reduced activity, and reduced motivation.

### Setting

The study will be conducted in the Third Affiliated Hospital of Sun Yat-sen University, a large comprehensive hospital in Guangzhou, China. The hospital is a tertiary medical facility that serves as a teaching hospital for Sun Yat-sen University, including 65 anesthesiologists in the Department of Anesthesiology, and the age of the front-line clinicians is 25 to 45. Besides, each anesthesiologist only undergoes one night shift each arm and works from 17:30 to 8:00 of the next day.

### Ethical approval and registration

The study protocol will be carried out in accordance with the principles of the Declaration of Helsinki, approved by the Institutional Review Board (IRB) of the hospital (approval number: [2020] 02-021-01), and registered with the Chinese Clinical Trial Registry at www.chictr.org on 21 March 2020 (registration no. ChiCTR2000031025). All the items of the WHO Trial Registration Data Set (TRDS) can be found in the protocol. The trial is currently active and ongoing, and any amendments to the protocol can be reported to and approved by the IRB. The participants can voluntarily withdraw from the study after providing informed consent.

### Inclusion and exclusion criteria

To be eligible, participants must meet all of the following inclusion criteria: (1) participants who work for the hospital, (2) aged 25–45 years, (3) the patients they are managing are class I or class II according to the American Society of Anesthesiologists (ASA), and (4) each operation takes 2 to 4 h. The exclusion criteria are as follows: (1) participants who refuse to sign the informed consent form, (2) participants with serious cardiopulmonary diseases, and (3) participants who had experienced physical discomfort, such as seizures, severe dizziness, eye twitching, or blackouts triggered by light flashes when they wore a VR headset.

### Randomization

Following informed consent, participants will be randomized in a 1:1 allocation to a sequence of either immersion relaxation using VR in the first period, followed by no immersion VR in the second treatment period, or no immersion VR in the first period, followed by immersion relaxation using VR in the second period, based on a computer-generated random number that is concealed via a sealed envelope. One of the researchers conducts this step. The allocation will not be disclosed to the other researchers who conduct the trial until the participant is enrolled and assigned. In addition, researchers who perform the statistical analyses will be blinded to the group allocation.

### Intervention group: virtual reality immersive relaxation

Participants in the VR immersion group (intervention group) will be asked to wear the VR headset (Pico G2 4K; Pico Technology Co., Ltd.; China) and choose the 360-degree panoramic videos on natural scenery around the world at https://vr.iqiyi.com/ based on their preferences to relax themselves in the resting room. In order to avoid selection bias stemming from the content of the videos, such as terrifying videos or rock songs, which may influence relaxation, the content of videos is limited to natural scenery. Considering the limitation of resting time in clinical practices, participants experience VR immersion for only 20 min for each time and they will be asked to watch videos for 20 min at 23:30 and at 07:30 of the next day. The timepoints are chosen based on our preliminary data that most first-line anesthesiologists would feel stress and overworked after 23:00, and the handover time of the night shifts (08:00 of the next day). In addition, according to our preliminary study, it took about 5 min to complete all the assessments at each timepoint. Thus, the participants are asked to complete the NASA-TLX, CPSS, VAS, and MFI-20 5 min before the specified timepoint. When the participants are watching the videos, anesthesiologists with the same qualifications will take over their work until the VR immersion is complete.

### Control group: the routine night-shift group

Participants in the control group will undergo a routine night shift without the VR immersion experience.

### Data collection

**Fig. 2:**
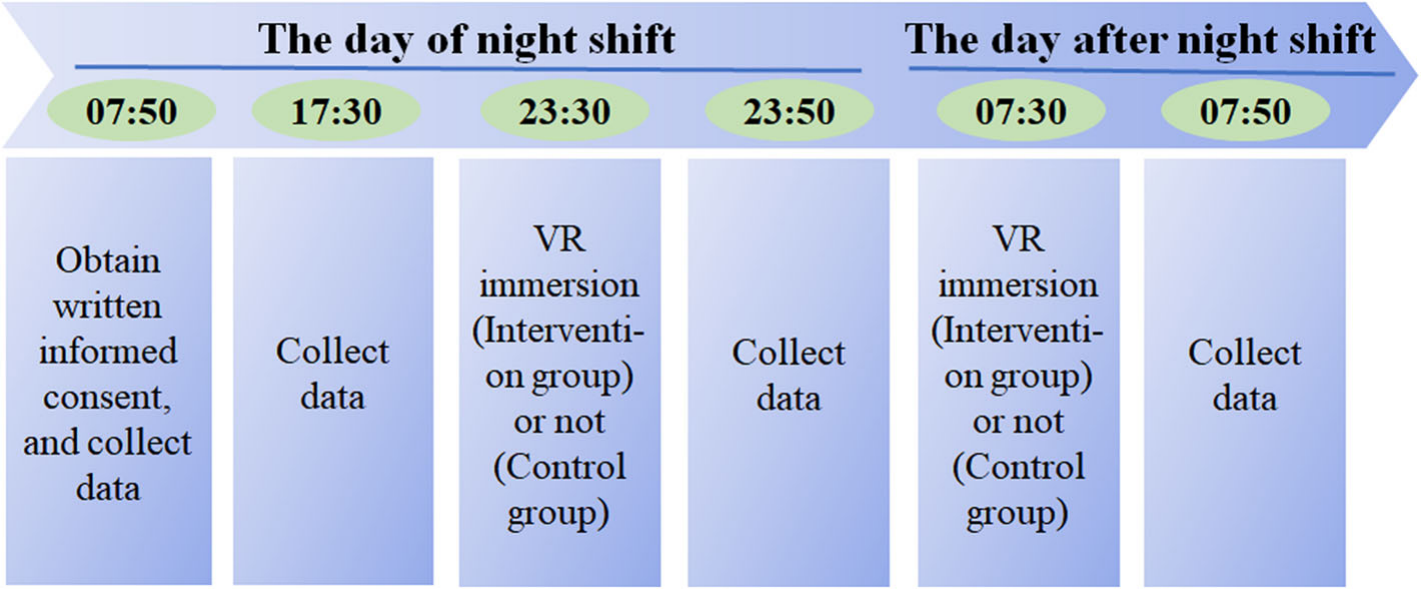
The data recording flow chart of the study

**Fig. 3:**
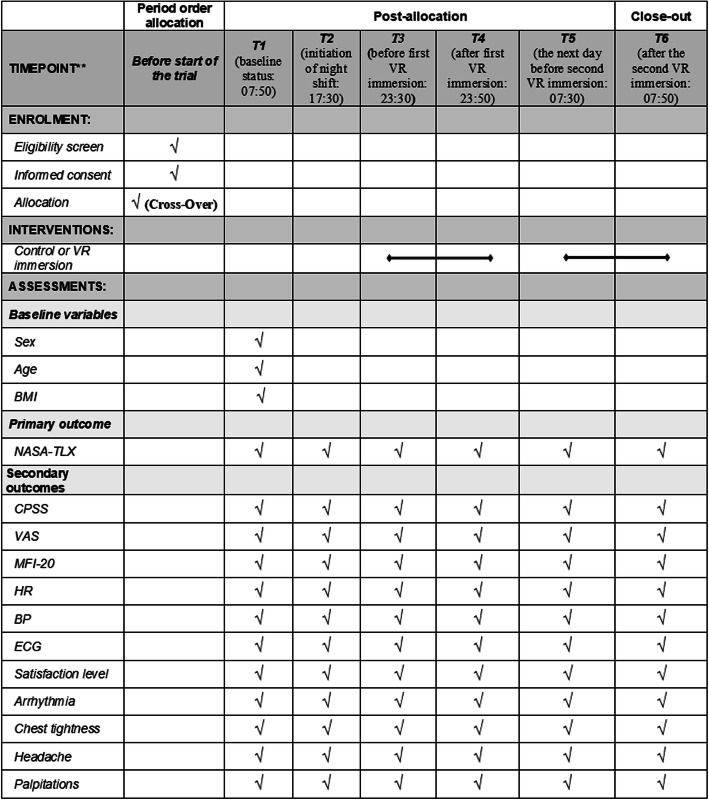
SPIRIT figure of the study

The participants’ general characteristics, including sex, age, body mass index (BMI), heart rate (HR), blood pressure (BP), and electrocardiogram (ECG) data, will be collected. To assess the effect of VR immersion on stress among night-shift anesthesiologists, data will be collected from participants in the intervention group at six timepoints: baseline status (07:50 am, T1), initiation of night shift (17:30, T2), before first VR immersion (23:30, T3), after first VR immersion (23:50, T4) on the day that they work the night shift and before second VR immersion (07:30, T5), and after first VR immersion (07:50, T6) of the next day. At each timepoint, participants will be asked to measure their stress status via completing the scales which included NASA-TLX, CPSS, VAS, and MFI-20. In addition, ECG will be measured using a mobile ECG recorder (CardioLearn; HeartVoice Medical Technology Co., Ltd., Anhui, China) at each timepoint following the manufacturer’s instructions. The mobile device has been widely used in China to simply measure the ECGs and automatically analyze their results based on a novel deep learning-based cloud service as earlier reported [27, 28]. Furthermore, the anesthesiologists will be invited to record their ECG whenever they feel discomfort during the night shift. The incidence of arrhythmia, level of satisfaction, and incidence of chest distress, headache, or heart palpitations will also be recorded by the researchers. The data recording flow chart is presented in Fig. 2 and the SPIRIT figure is shown in Fig. 3.

Two researchers blinded to the grouping are responsible for data entry and reconciliation. Both the paper and electronic data are retained by the corresponding authors, and the data will be kept forever after the trial is completed.

### Reporting of compliance and adverse events

To avoid possible bias, participants who had experienced something very pleasant or sad during the washout time will be asked to report their own emotion status before the second enrollment. This would be recorded and judged by two independent psychologists and those who get married or promoted, whose child is born, or whose family member is dead would be excluded from the final analysis. It has been reported that young people are more vulnerable to physical discomfort, such as seizures, severe dizziness, eye twitching, or blackouts triggered by light flashes when wearing a VR headset [29]; therefore, for participants who experience any discomfort during the VR immersion procedure, the trial can be stopped, and the participants will be treated accordingly. All these events are recorded and reported. To ensure the safety of patients, anesthesiologists with the same qualifications will take over the participants’ work during the VR immersion.

### Statistical analysis

#### Sample size estimation

The sample size for this study was estimated using the PASS program 15.0.5. Based on our preliminary data from 14 subjects, the mean ± SD of the NASA-TLX was 65.5 ± 16.8 before the intervention and 55.7 ± 18.2 after the intervention, and the SD of the difference was 12.1. According to tests for the difference between two means in a 2 × 2 crossover design, assuming a two-sided *α* of 0.05 and a statistical power of 80%, 26 participants are needed. To account for a potential dropout rate of 10%, we aim to recruit a sample size of 30 participants in total, and thus, 15 participants are needed for each sequence.

#### Data analysis

All data analysis will be performed using SPSS for Windows V.16.0 (SPSS Inc., Chicago, IL, USA). To avoid bias, the data analyst will be blinded to the data. The per-protocol analysis will be performed in the current study. Participants who successfully complete the study according to the intervention plan would be included in the analysis. The Kolmogorov-Smirnov and Shapiro-Wilk tests will be used to test the normality of continuous data. Normally distributed data will be presented as the mean ± SD, and nonnormally distributed data will be expressed as the median (interquartile range). All quantitative data will be analyzed using methods applicable to between-group (intervention group compared with the control group), within-group (post-intervention data compared with pre-intervention data within the intervention group and the control group, respectively), and between-consequence comparisons. Differences are considered significant when a two-sided *P* value is less than 0.05.

#### Analysis of the primary outcome

Our primary outcome, the score of the NASA-TLX, will be compared between the two groups, two periods, and two consequences using one-way analysis of variance for the 2 × 2 crossover design.

#### Analysis of the secondary outcomes

The effect of VR immersion on the CPSS, VAS, and MFI-20 scores will be compared using one-way analysis of variance for the 2 × 2 crossover design. Spearman’s rank correlation test will be used to analyze the association between work hours and scores on the scales. The age, BMI, HR, MBP, and the level of satisfaction between groups will be analyzed by the Student *t* test or the Wilcoxon rank-sum test. The gender, incidence of arrhythmia, chest distress, headache, and heart palpitations between groups will be analyzed by the Pearson *χ*^2^ test or Fisher’s exact test.

## Discussion

This study will be the first to evaluate the potential effectiveness of VR technology in reducing stress among night-shift anesthesiologists. Work overload, long working hours, and continuous noise pollution [30, 31] are among the factors that cause physical and mental problems among anesthesiologists, such as psychological distress, burnout syndrome (BOS), and memory and attention problems [32, 33]. It has been reported that mental fatigue is the main cause of medical error among anesthesiologists [34] as well as the main reason for the high proportion of suicides among these doctors [35]. A study showed that the risk of medical accidents increases exponentially with each hour of work after the doctor has worked for 9 h consecutively. Moreover, the impairment of psychomotor function may be equivalent to a blood alcohol concentration of 0.1% if the doctor has experienced 24 h of sustained wakefulness; this concentration is higher than the legal limit for driving in most states in the USA [36]. It has also been reported that the rate of burnout among anesthesiologists is 48%, higher than that among all physicians among the specialties studied [37]. The burnout rate among Chinese anesthesiologists is 69%, while their consultation rates are 73% [38]. It is an urgent necessity to take more measures to curb psychological distress without increasing the number of anesthesiologists.

VR technology has been widely used in clinical practice to treat mental illnesses and reduce preoperative anxiety in both healthcare providers and patients [16, 17, 19]. Shah et al. [39] found that stress was the primary target and depression was the secondary target in the VR mood induction procedure study. The current study would provide evidence affirming the use of VR technology to reduce stress among night-shift anesthesiologists. These results might be attributed to the fact that watching VR videos could reduce the long hours of night-shift work, distract the anesthesiologists, and allow them to rest, thus making them positive and vibrant again.

However, there are still several limitations in the study. First, because of the connectivity issues of VR technology, a nonimmersive environment may arise, which may interfere with participant relaxation and the subsequent level of satisfaction. Second, in the current study, to better ensure the safety of patients, we will arrange another extra qualified anesthesiologist to take over the participants’ work during the VR immersion. However, this might be unlikely in the real-world where extra anesthesiologists are not always present. We hope the results of the study may spur managers to take measures to enable the anesthesiologists to enjoy VR entertainment during the night shift in the future. Third, the study is a single-center, crossover design with a small sample size. Therefore, it is better to confirm the preliminary results by conducting a large-scale multicenter study.

Although VR technology has been widely used in biomedical fields, it is still unknown whether the use of VR can reduce stress among night-shift anesthesiologists during working hours. A positive result in this trial could spur hospitals to apply the technology to reduce stress among night-shift doctors in every department, thus creating a relatively relaxed working environment that can benefit both doctors and patients.

## Data Availability

Data of this study are available on reasonable request from the corresponding authors. The data is monitored by the corresponding authors and scientific research department of the hospital.

## Abbreviations

ASA: American Society of Anesthesiologists
BOS: Burnout syndrome
BMI: Body mass index
CPSS: Chinese Perceived Stress Scales
CRH: Corticotropin-releasing hormone
CRP: C-reactive protein
ECG: Electrocardiogram
HR: Heart rate
IRB: Institutional Review Board
MBP: Mean blood pressure
MFI-20: Multidimensional Fatigue Inventory
NASA-TLX: NASA Task Load Index
PTSD: Posttraumatic stress disorder
VAS: Visual analogue scale
VR: Virtual reality
